# Unconditional and conditional QTL analyses of seed fatty acid composition in *Brassica napus* L.

**DOI:** 10.1186/s12870-018-1268-7

**Published:** 2018-03-23

**Authors:** Feng Chen, Wei Zhang, Kunjiang Yu, Lijie Sun, Jianqin Gao, Xiaoying Zhou, Qi Peng, Sanxiong Fu, Maolong Hu, Weihua Long, Huiming Pu, Song Chen, Xiaodong Wang, Jiefu Zhang

**Affiliations:** Provincial Key Laboratory of Agrobiology, Key Laboratory of Cotton and Rapeseed, Ministry of Agriculture, Institute of Industrial Crops, Jiangsu Academy of Agricultural Sciences, Nanjing, China

**Keywords:** *Brassica napus* L., Fatty acid, Conditional QTL, Unconditional QTL

## Abstract

**Background:**

The fatty acid composition of *B. napus*’ seeds determines the oil’s nutritional and industrial values, and affects seed germination. Many studies have reported correlations among C16:0, C18:0, C18:1, C18:2 and C18:3 based on phenotypic data; however, the genetic basis of the fatty acid composition in *B. napus* is still not well understood.

**Results:**

In this study, unconditional and conditional quantitative trail locus (QTL) mapping analyses were conducted using a recombinant inbred line in six environments. In total, 21 consensus QTLs each for C16:0, C18:0 and C18:2, 16 for C18:1 and 22 for C18:3 were detected by unconditional mapping. The QTLs with overlapping confidence intervals were integrated into 71 pleiotropically unique QTLs by meta-analysis. Two major QTLs, *uuqA5–6* and *uuqA5–7*, simultaneously affected the fatty acids, except C18:0, in most of environments, with the homologous genes fatty acid desaturase 2 (*FAD2*) and glycerol-3-phosphate sn-2-acyltransferase 5 (*GPAT5*) occurring in the confidence interval of *uuqA5–6*, while phosphatidic acid phosphohydrolase 1 (*PAH1*) was assigned to *uuqA5–7*. Moreover, 49, 30, 48, 60 and 45 consensus QTLs were detected for C16:0, C18:0, C18:1, C18:2 and C18:3, respectively, by the conditional mapping analysis. In total, 128 unique QTLs were subsequently integrated from the 232 conditional consensus QTLs. A comparative analysis revealed that 63 unique QTLs could be identified by both mapping methodologies, and 65 additional unique QTLs were only identified in conditional mapping.

**Conclusions:**

Thus, conditional QTL mapping for fatty acids may uncover numerous additional QTLs that were inhibited by the effects of other traits. These findings provide useful information for better understanding the genetic relationships among fatty acids at the QTL level.

**Electronic supplementary material:**

The online version of this article (10.1186/s12870-018-1268-7) contains supplementary material, which is available to authorized users.

## Background

*Brassica napus* (AACC, 2n = 38) is the second most important oilseed crop worldwide. *B. napus*’ oils have diverse uses, ranging from food to industrial feedstock, and are an environmentally friendly and renewable energy source [1]. Fatty acid (FA) composition significantly affects the function, quality and nutritional properties of vegetable oils. To meet the steadily growing global requirements for rapeseed oil, there is an urgent need to develop desirable cultivars with improved FA compositions.

Generally, the modern rapeseed varieties produce oil with less than 2% erucic acid, 5% to 8% saturated fats (mainly palmitic and stearic acids), 60% to 65% monounsaturated fats (mainly oleic acid) and 30% to 35% polyunsaturated fats (mainly linoleic and linolenic acids) [2]. Seed’ FA compositions in *B. napus* are quantitative traits controlled by multiple genes and affected by environmental factors [3]. QTL mapping is the preliminary step toward dissecting the genetic mechanisms of these complex traits, and a number of QTLs affecting different FAs were identified over the past 20 years. For the two saturated FA components, the major QTLs for palmitic acid (C16:0) have mostly been identified on linkage groups A8, A10, C1, C4 and C8 in previous studies [3–7]; and the major QTLs for stearic acid (C18:0) are generally located on A1, A5, A6, A7, A8 and C3 [3–7]. Many studies focused on oleic acid (C18:1), and the major QTLs are mainly distributed across A3, A5, A8, C3 and C8 [3–10]. For the two polyunsaturated fats, the major QTLs for linoleic acid (C18:2) are largely across A5, A8, A9, C3 and C4 [3–9], and the major QTLs for linolenic acid (C18:3) are mainly distributed on A4, A5, A6, A7, C3 and C4 [3–10].

Although several major QTLs have been identified for seed FA composition in *B. napus*, few of them could be effectively utilized in breeding programs because most of the studies have been based on low-density genetic maps and applied traditional markers, resulting in QTLs with large confidence intervals. High-density maps could benefit QTL mapping by providing more precise parameter estimates [11]. In *B. napus*, the Brassica 60 K single nucleotide polymorphism (SNP) BeadChip Array, containing 52,157 SNP loci, was produced [12, 13], which has facilitated the construction of a high-density, sequence-based, genome-wide polymorphism screening map. Several high-density SNP maps were constructed to identify agronomically important traits, such as seed fiber [14], boron efficiency [15], apetalous characteristics [16] and seed oil and protein contents [17, 18]. Using high-density SNP markers, loci for the FA composition of *B. napus* were detected in both QTL mapping [19] and genome-wide association studies (GWAS) [20, 21].

FA biosynthesis in plants is a very complicated process. In *Arabidopsis*, more than 600 genes encoding enzymes or regulatory factors are involved in acyl-lipid metabolism [22]. However, only approximately 20% of these genes are represented by defined and characterized mutants [22]. The allotetraploid *B. napus* has a close evolutionary relationship with *Arabidopsis* [23, 24]. Although the biological pathways of FA biosynthesis and modifications are well documented in *Arabidopsis*, lipid metabolism and its regulation are less well understood in *B. napus.* Different FA compositions share the same basic resources and are controlled by the same FA synthesis-related genes in plastids [22]. In most studies, different FAs are correlated with each other based on phenotypic data, and many of the QTLs for different FAs are co-localized [3–6, 9]. When this occurs, it is difficult to distinguish such loci with pleiotropic effects from different tightly linked genes underlying the same locus or the specific genes control multiple traits [25]. A method for the multivariable conditional analysis was proposed for determining the contributions of component traits to a complex trait and for investigating the genetic relationship between two traits at the QTL level [26, 27]. The conditional analysis method could exclude the contribution of a causal trait to the variation of the resultant trait [28]. Using the C16:0 and C18:1 content as an example, C18:1 conditioning on C16:0 allows a C18:1 analysis to be conducted independently of variation in C16:0 if C18:1 is genetically correlated with C16:0. The major advantage of this method is that the net contribution of C16:0 to C18:1 could be effectively determined. Based on this methodology, the genetic relationships between putatively interrelated traits in crops, such as plant height with respect to spike and internode lengths in wheat [29] and grain yield and its component traits in rice [30]. In *B. napus*, Zhao et al. [31] performed an interrelationship analysis between oil and protein contents, and found six QTLs had pleiotropic effects on both traits. However, none of the studies considered the FA composition in *B. napus*’ seeds.

In this paper, a recombinant inbred line (RIL) was used to investigate the genetic relationships among C16:0, C18:0, C18:1, C18:2 and C18:3 in six experiments. The objectives were to: (1) identify QTLs affecting the FA composition of *B. napus*’ seeds using a high-density SNP map; and (2) specify the genetic relationships among FAs at the QTL level by utilizing unconditional and conditional mapping approaches. The research will contribute to a better understanding of the genetic architecture of the FA composition in *B. napus*’ seeds.

## Results

### Phenotypic variation and correlation analysis for FA compositions

**Fig. 1:**
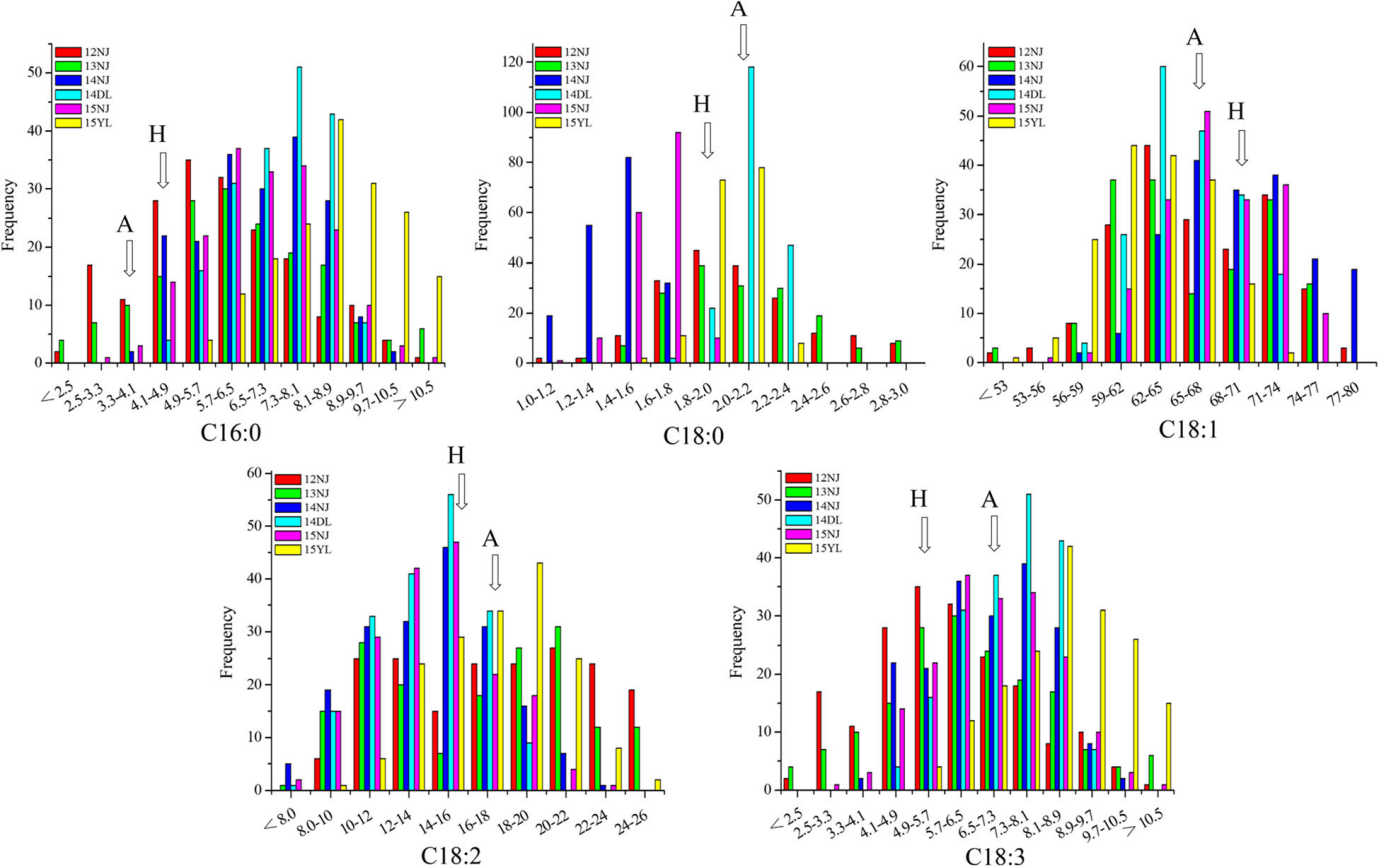
Phenotypic variation of the five fatty acid composition in the AH RIL population. The *x*-axis represents the percentage of the five FA content, and the *y*-axis represents the number of lines. FAs in different experiments were discriminated using different colored boxes (12NJ, red; 13NJ, green; 14NJ, blue; 14DL, cyan; 15NJ, magenta; 15YL, yellow). A represents the female parent “APL01” and H represents the male parent “Holly” of the AH population

The phenotypic values of C16:0, C18:0, C18:1, C18:2 and C18:3 for the AH population were measured in six experiments. There was a wide segregation range for the five FA compositions, with a continuous normal distribution in all trials (Fig. 1), indicating that the compositions were all quantitative traits controlled by polygenes. Strong transgressive segregations were observed in all experiments (Fig. 1).

Table 1 shows the correlation coefficients between different FA compositions based on means of AH lines in Shannxi and Jiangsu Provinces. C16:0 showed a highly positive correlation with C18:0, C18:2 and C18:3, but had a significant negative relationship with C18:1 in both locations. C18:1 is the most important unsaturated FA in the oil based on potential human health effects, and it had a significant negative relationship with the other FA levels, except C18:0.

**Table 1:** Phenotypic correlations among five fatty acid compositions in Shannxi (above diagonal) and Jiangsu (below diagonal) Provinces

	C16:0	C18:0	C18:1	C18:2	C18:3
C16:0	1	0.35^**^	−0.81^**^	0.81^**^	0.69^**^
C18:0	0.29^**^	1	0.03	−0.06	− 0.11
C18:1	−0.68^**^	0.01	1	−0.98^**^	−0.96^**^
C18:2	0.71^**^	0.02	−0.90^**^	1	0.94^**^
C18:3	0.38^**^	−0.10	−0.67^**^	0.46^**^	1

^**^Represents statistical significance at *P* = 0.01

### Unconditional QTL analysis of five FA compositions

**Fig. 2:**
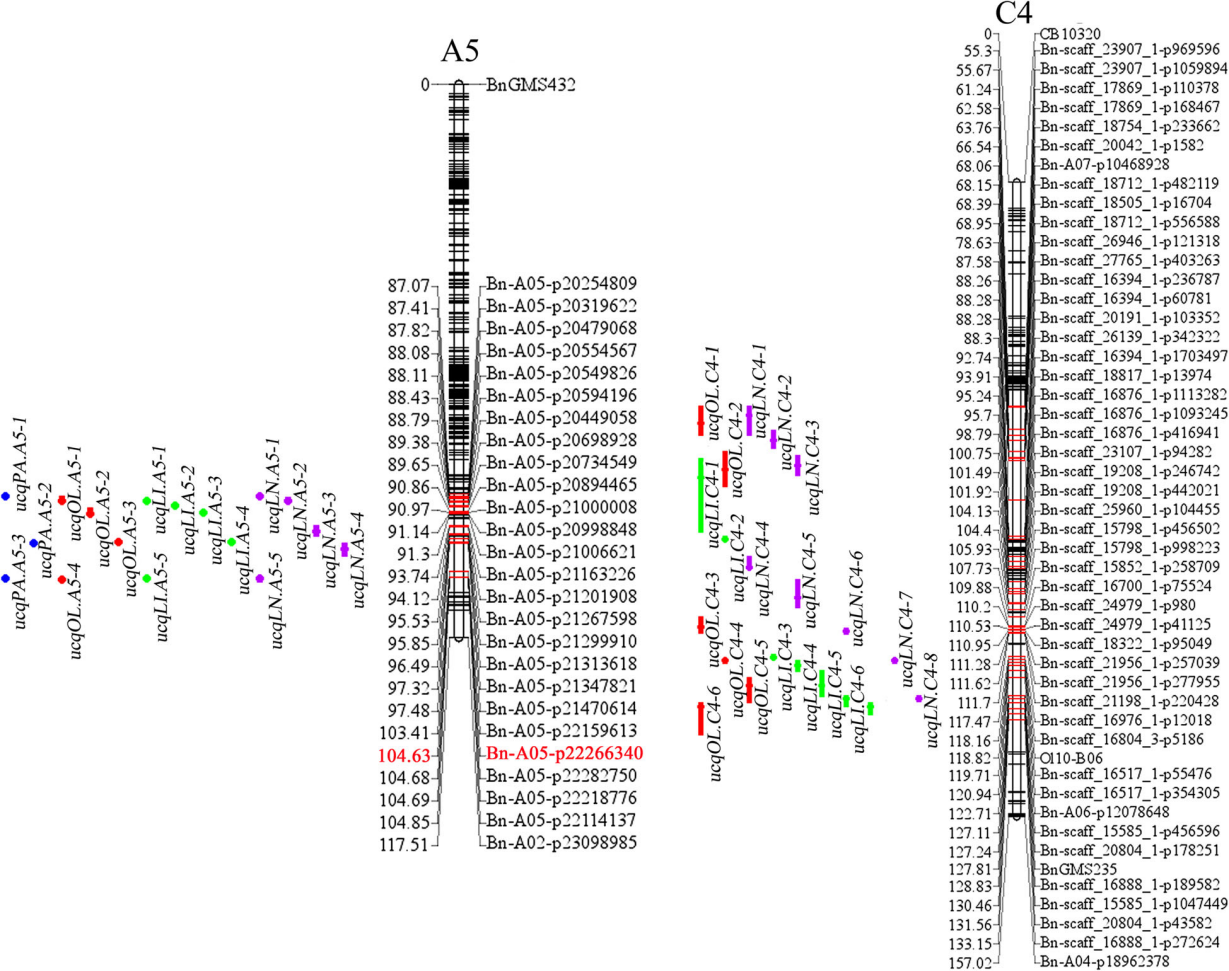
The locations of consensus QTLs for fatty acids identified by unconditional QTL mapping analysis. The loci names and the positions are listed on the right and the left of the linkage groups, respectively. For simplicity, only the markers underlying the QTL’ CIs and the terminal two markers of each linkage group are shown. The loci that were not underlying QTL’ CIs are only labeled with black short bars. QTLs for different traits are discriminated using different color bars (blue bar, C16:0; red bars, C18:1; green bars, C18:2; purple bars, C18:3)

For C16:0, 33 identified QTLs were detected across the six environments (Additional file 1). Among them, 15 QTLs co-localized on A5, and they were integrated into three consensus QTLs, *ucqPA.A5–1*, *ucqPA.A5–2* and *ucqPA.A5–3* (Fig. 2). The remaining 18 QTLs were only detected on one specific environment and were considered consensus QTLs, thereby resulting in a total of 21 consensus QTLs. Two major QTLs, *ucqPA.A5–2* and *ucqPA.A5–3*, stably expressed in all of the six environments, and explained 12.14%–42.96% and 11.11%–28.71% of PV, respectively (Additional file 1).

For C18:0, 26 identified QTLs were resolved (Additional file 2). Each QTL accounted for 4.42%–17.40% of PV, with five individually explaining more than 10% of PV. These QTLs were integrated into 21 consensus QTLs, including 1 consensus QTL, *ucqST.C3–2*, which was detected in three environments, 3 QTLs were detected in two environments, and 17 QTLs that were only expressed in a single environment (Additional file 2). The QTL *ucqST.C3–2*, with PV ≥ 10% in 14DL, 14NJ and 15NJ (16.35%, 17.40% and 15.24%, respectively), was considered the major QTL.

For C18:1, between 3 and 7 identified QTLs were detected in single environment, and 30 identified QTLs were obtained over the six tested environments (Table 2). These QTLs contributed 3.52%–49.55% of PV. Among them, 18 overlapping QTLs formed QTL clusters on A5 and C4 chromosomes and were integrated into 4 consensus QTLs, *ucqOL.A5–3*, *ucqOL.A5–4*, *ucqOL.C4–2* and *ucqOL.C4–4* (Fig. 2). Twelve other identified QTLs were only expressed in one environment. The *ucqOL.A5–3* and *ucqOL.A5–4* were the two major QTLs, which were repeatedly detected in all six environments, explaining from 19.83% to 49.55% and from 13.70% to 35.97% of the PV, respectively (Table 2). Two QTLs, *ucqOL.A5–1* and *ucqOL.A5–2*, were only detected in 13NJ and 14DL, although they accounted for as much as 25.52% and 30.11% of the PV, respectively.

**Table 2:** Unconditional identified QTLs and consensus QTLs obtained for C18:1 in six environments

Unconditional Consensus QTL			Unconditional Identified QTL							
QTL	Peak	CI.	QTL^a^	Chr.^b^	Position	LOD	Additive	PV^c^	CI.^d^	Env.^e^
*ucqOL.A4*	30.81	30.2–31.6	*uiqOL5.A4*	A4	30.81	3.80	−1.00	5.55	30.2–31.6	15NJ
*ucqOL.A5–1*	88.41	87.4–88.8	*uiqOL2.A5–1*	A5	88.41	12.2	−3.13	25.52	87.4–88.8	13NJ
*ucqOL.A5–2*	91.21	90.1–91.3	*uiqOL4.A5–1*	A5	91.21	17.64	−2.01	30.11	90.1–91.3	14DL
*ucqOL.A5–3*	97.22	96.86–97.57	*uiqOL5.A5–1*	A5	96.51	17.17	−2.34	29.95	95.9–97.5	15NJ
			*uiqOL2.A5–2*	A5	97.31	28.63	−4.36	49.22	96.5–99.9	13NJ
			*uiqOL3.A5–1*	A5	97.31	31.64	−3.62	49.55	96.5–97.5	14NJ
			*uiqOL6.A5–1*	A5	97.31	10.57	−1.86	19.83	96–97.5	15YL
			*uiqOL1.A5–1*	A5	99.51	17.74	−3.74	33.63	97.5–103.3	12NJ
			*uiqOL4.A5–2*	A5	99.51	29.9	−2.59	49.39	97.3–102.7	14DL
*ucqOL.A5–4*	105.17	104.66–105.68	*uiqOL1.A5–2*	A5	104.61	11.53	−2.83	19.69	103.4–106.9	12NJ
			*uiqOL2.A5–3*	A5	104.91	18.63	−3.72	35.97	104.8–108.5	13NJ
			*uiqOL4.A5–3*	A5	104.91	15.17	−1.89	26.63	104.6–106.9	14DL
			*uiqOL3.A5–2*	A5	104.91	16.93	−2.82	30.69	104.6–106.9	14NJ
			*uiqOL6.A5–2*	A5	104.91	7.05	−1.54	13.70	104.6–107.2	15YL
			*uiqOL5.A5–2*	A5	105.91	9.04	−1.89	19.85	105.4–107.3	15NJ
*ucqOL.A9–1*	94.51	94.2–95.8	*uiqOL4.A9*	A9	94.51	3.64	0.73	4.02	94.2–95.8	14DL
*ucqOL.A9–2*	96.31	95.6–97.2	*uiqOL6.A9*	A9	96.31	2.91	0.95	5.26	95.6–97.2	15YL
*ucqOL.C1*	22.51	16.4–23.8	*uiqOL3.C1*	C1	22.51	3.29	0.98	3.62	16.4–23.8	14NJ
*ucqOL.C3*	97.51	96.6–98.2	*uiqOL1.C3*	C3	97.51	3.16	1.66	4.46	96.6–98.2	12NJ
*ucqOL.C4–1*	59.71	55.4–62.6	*uiqOL4.C4–1*	C4	59.71	3.17	−0.71	3.77	55.4–62.6	14DL
*ucqOL.C4–2*	71.01	66.65–75.36	*uiqOL4.C4–2*	C4	71.01	5.34	−0.97	7.09	65.5–77.4	14DL
			*uiqOL3.C4–1*	C4	71.01	3.86	−1.13	4.93	68.1–80.9	14NJ
*ucqOL.C4–3*	110.21	107.7–111.7	*uiqOL6.C4–1*	C4	110.21	2.92	−0.94	5.07	107.7–111.7	15YL
*ucqOL.C4–4*	118.46	117.86–119.06	*uiqOL6.C4–2*	C4	118.21	4.54	−1.17	7.74	117.5–118.8	15YL
			*uiqOL3.C4–2*	C4	119.71	3.97	−1.09	4.39	118.8–125.7	14NJ
			*uiqOL5.C4–1*	C4	119.71	4.75	−1.13	7.08	118.8–122.7	15NJ
			*uiqOL4.C4–3*	C4	121.01	3.29	−0.70	3.52	118.8–126.5	14DL
*ucqOL.C4–5*	124.71	122.7–128.8	*uiqOL5.C4–2*	C4	124.71	3.99	−1.10	6.61	122.7–128.8	15NJ
*ucqOL.C4–6*	129.81	128.8–137	*uiqOL5.C4–3*	C4	129.81	4.04	−1.07	6.49	128.8–137	15NJ
*ucqOL.C6*	27.61	22.3–29.8	*uiqOL3.C6*	C6	27.61	3.58	−1.01	3.88	22.3–29.8	14NJ

*DL* Dali, *YL* Yangling, *NJ* Nanjing, *12*, *13*, *14* and *15* denote the years 2012, 2013, 2014 and 2015, respectively

^a^Identified QTLs detected in different experiments

^b^Chromosome

^c^The phenotypic variation explained by the identified QTL

^d^The 2-LOD confidence interval of QTLs

^e^The experiment in which the QTLs were detected

For C18:2, 37 identified QTLs were found in six environments, with the contributions of individual QTL ranging from 3.01% to 59.21% (Additional file 3). After integrating these overlapping QTLs, 21 consensus QTLs were obtained, including 15 QTLs that were detected only in single environments. Of these, two QTLs, *ucqLI.A5–4* and *ucqLI.A5–5*, accounting for 19.49%–59.21% and 13.58%–41.37%, respectively, of the PV, were detected in all six environments and inferred to be major QTLs (Fig. 2). Interestingly, QTLs *ucqLI.A5–1* and *ucqLI.A5–3* were only detected in 12NJ and 13NJ, although they explained 23.61% and 32.41% of the PV, respectively.

For C18:3, 36 identified QTLs were detected in six environments. They explained 2.80%–46.32% of the PV in each trial (Additional file 4). After the meta-analysis, 22 consensus QTLs were obtained, including 6 integrated from 21 identified QTLs with overlapping CIs and 15 non-overlapping QTLs. Each single QTL was repeatedly detected in five (*ucqLN.C4–7*) and four (*ucqLN.A5–5*) of the environments, and explained 2.71%–23.99% and 3.65%–33.37% of the PV, respectively. In addition, *ucqLN.A5–2* and *ucqLN.A5–3* each explained more than 20% of the PV and were detected only in single environments.

### Unconditional unique QTL for the five traits

In total, 101 consensus QTLs for the five examined traits were obtained, including 21 each for C16:0, C18:0 and C18:2, 16 for C18:1 and 22 for C18:3. A large proportion of the QTLs formed clusters on several chromosomal regions, indicating that these loci might affect several FA contents. To distinguish genetic explanations of the correlations between the FA concentrations, these consensus QTLs were integrated into unique QTLs. Consequently, 71 unique QTLs distributed throughout 17 chromosomes (excluding A10 and C8) were obtained, with the main QTLs controlling one (49 QTLs) or two (16 QTLs) traits (Additional file 5). Four unique QTLs (*uuqA4–2*, *uuqA5–2*, *uuqC4–3* and *uuqC4–9*) simultaneously affected C18:1, C18:2 and C18:3. All four of these unique QTLs had positive additive effects on C18:2 and C18:3, but had negative additive effects on C18:1. Furthermore, two QTLs (*uuqA5–6* and *uuqA5–7*) controlled the FA contents, except C18:0, were scattered over the A5 chromosome, with very close distances, and contributed a large proportion of PV for each FA content in most of the environments. Both QTLs had positive additive effects on C16:0, C18:2 and C18:3, but had significant negative additive effects on C18:1. These findings may explain the high positive correlations between C16:0, C18:2 and C18:3, and their remarkable negative correlations with C18:1 and weak correlations with C18:0, as shown in Table 1.

### Conditional QTL analysis for five fatty acid compositions

**Fig. 3:**
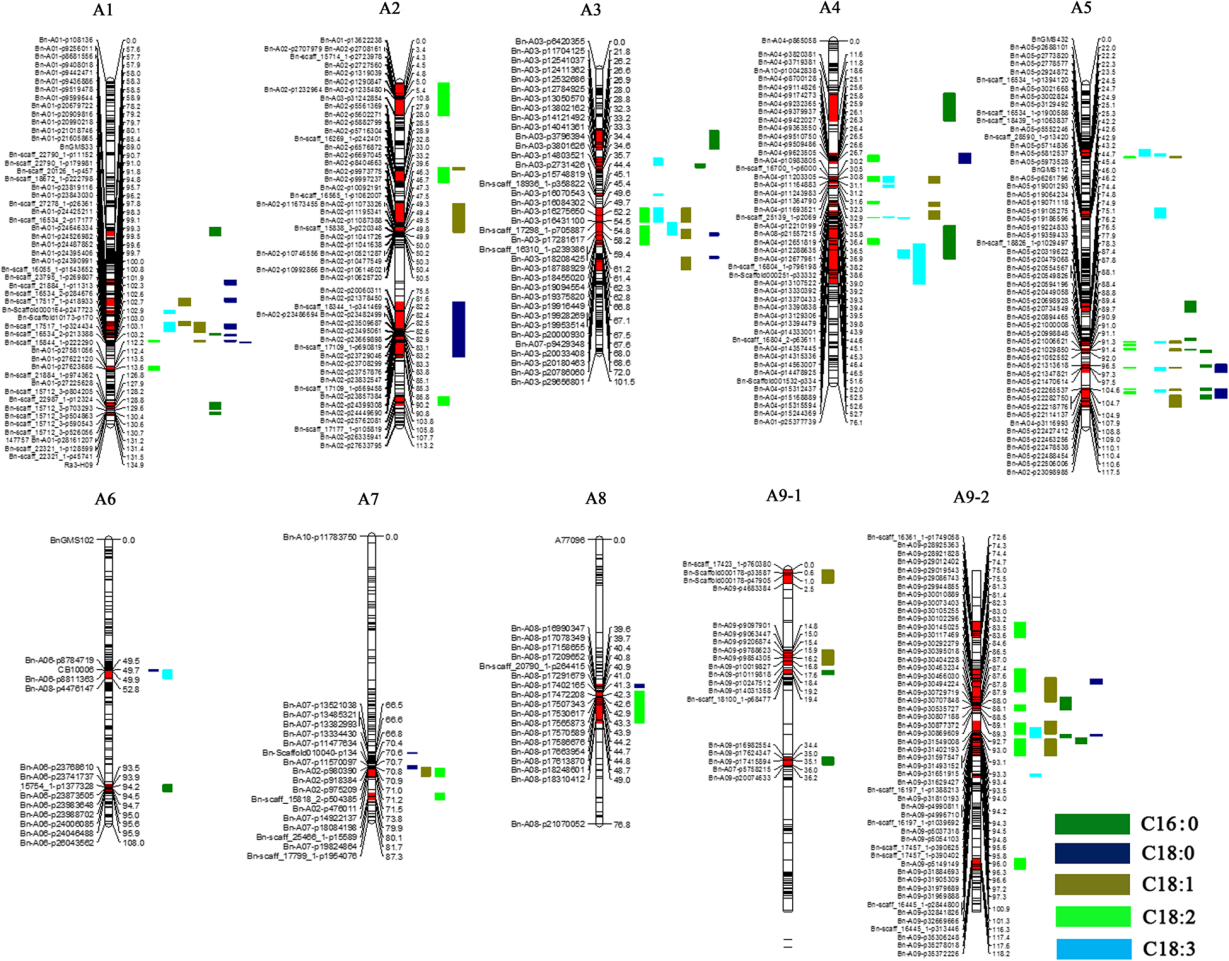
The locations of conditional consensus QTLs associated with fatty acids in the AH map. Conditional consensus QTLs distributed across the A subgenome are shown in this figure, and QTLs on the C subgenome are supplied in Additional file 7. The linkage groups are represented by vertical bars. The locus name and genetic distance are listed on the right and left of the corresponding chromosomes, respectively. The red regions on the linkage groups indicate that these regions harbor QTLs identified by the conditional QTL mapping analysis. Different colors denote different traits as indicated in the bar shown at the lower right corner of the picture

**Fig. 4:**
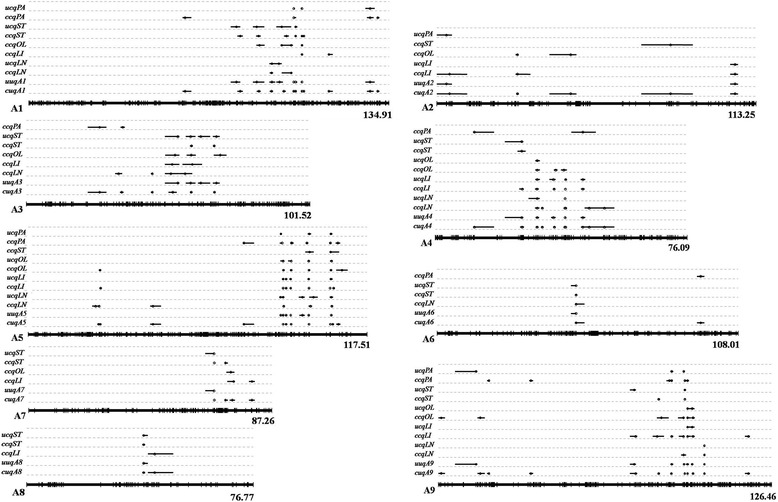
QTL comparison of the five fatty acid concentrations between unconditional and conditional mapping methodologies. QTLs located on the A subgenome are shown in this figure, and QTLs mapped to the C subgenome are provided in Additional file 8. Whole linkage groups are shown with black lines on the bottom, and molecular markers are labeled with short vertical bars. Consensus QTLs and unique QTLs obtained by the two methods are compared, and the QTL nomenclature is based on the descriptions in the Materials and methods (for example, *ucqPA* means unconditional consensus QTLs for C16:0). The black lines above the linkage groups show the QTL’ CIs, and the circles indicate the peak positions

When C16:0 was conditioned on C18:0, C18:1, C18:2 and C18:3, 39, 19, 29 and 29 identified conditional QTLs, respectively, were detected in the six environments (Additional file 6). The PV explained by each QTL ranged from 3.05% to 39.16%. A meta-analysis of these 116 identified QTLs resulted in 49 consensus QTLs, including 28 consensus QTLs that were formed by integrating 95 overlapping identified QTLs (Fig. 3, Additional files 6 and 7). Comparing the two mapping methodologies, 18 unconditional QTLs were co-localized with the conditional QTLs (Fig. 4, Additional files 8 and 9). The two major unconditional QTLs, *ucqPA.A5–2* (co-localized with *ccqPA.A5–5*) and *ucqPA.A5–3* (co-localized with *ccqPA.A5–6*), still showed additive effects with similar values to the corresponding conditional QTLs when the influence of C18:0 on C16:0 was excluded (Additional files 1 and 6). Moreover, the conditional QTL mapping of C16:0 uncovered 31 new QTLs (Additional file 9).

Conditional QTL mapping for C18:0 detected 129 identified QTLs in the six environments, including 25, 37, 32 and 35 QTLs in the conditions of ST/PA, ST/OL, ST/LI and ST/LN, respectively (Additional file 10). These QTLs were distributed on 14 chromosomes (Fig. 3 and Additional file 7), and a single QTL was responsible for 4.08%–18.45% of the total PV. In addition, the 129 identified QTLs were integrated into 30 consensus QTLs. Further analyses showed that 16 conditional consensus QTLs were conserved by unconditional consensus QTLs (Fig. 4, Additional files 8 and 11). C18:0 showed significant positive correlations with C16:0 (Table 1), and several QTLs failed to show significant effects when the influence of C16:0 on C18:0 was excluded in a specified environment, such as *ccqST.A5–1*, *ccqST.A6*, *ccqST.A9–2* or *ccqST.C5–2* (Additional file 10).

Conditional QTL mapping for C18:1 uncovered 109 identified QTLs under six environments, with 32 for OL/PA, 33 for OL/ST, 19 for OL/LI and 25 for OL/LN (Additional file 12). These QTLs were distributed throughout 15 chromosomes, and accounted for 3.11%–48.53% of the PV (Fig. 3, Additional files 7 and 12). The 109 identified QTLs were integrated into 48 consensus QTLs. Among the 16 consensus QTLs detected by unconditional QTL mapping, 14 were conserved between the two mapping methodologies (Fig. 4, Additional files 8 and 13). For example, the two major unconditional QTLs, *ucqOL.A5–3* and *ucqOL.A5–4*, were located in the same CI as conditional QTLs *ccqOL.A5–4* and *ccqOL.A5–5*, respectively. When C18:1 was conditioned on C18:0 in all six environments, *ccqOL.A5–4* and *ccqOL.A5–5* showed similar additive effects and contributions to the PV compared with unconditional QTLs *ucqOL.A5–3* and *ucqOL.A5–4*, respectively (Additional files 2 and 12). Thus, the genes that influence C18:1 at the two loci were independent from the C18:0 content, and this is also consistent with the correlation analysis which showed that C18:1 has no significant relationship with C18:0 (Table 1). However, when C18:1 was conditioned on C18:2, *ccqOL.A5–4* and *ccqOL.A5–5* failed to show significant effects in all of the environments (Additional file 12), indicating that the same genes underlying the two loci affected the C18:1 and C18:2 contents, rather than different tightly linked genes.

QTL mapping for C18:2 conditioned on C16:0, C18:0, C18:1 and C18:3 showed 42, 42, 29 and 35 identified QTLs, respectively, with single QTLs explaining 2.03%–62.70% of the PV (Additional file 14). Among the abovementioned 148 QTLs, 115 QTLs had overlapping CIs and were integrated into 27 consensus QTLs. Thus, together with the 33 non-overlapping QTLs, 60 conditional consensus QTLs were obtained for C18:2 (Fig. 3 and Additional file 7). Among the 21 unconditional consensus QTLs for C18:2, 19 still had significant effects when C18:2 was conditioned on the four other FA contents, whereas 41 additional QTLs were only identified in the conditional mapping (Fig. 4, Additional files 8 and 15). Most of the additional QTLs showed minor effects, but several QTLs had significant effects, such as *ccqLI.A5–7* and *ccqLI.C9–2* (Additional file 14).

For C18:3, 144 conditional identified QTLs were detected in the six environments, accounting for 2.34%–51.0% of the PV, including 37 for LN/PA, 41 for LN/ST, 31 for LN/OL and 35 for LN/LI (Additional file 16). In total, 126 identified overlapping QTLs and 18 non-overlapping QTLs were subsequently integrated into 45 consensus QTLs. Comparing the results gained from the two mapping methodologies, 15 consensus QTLs were detected by both the unconditional and conditional analyses (Fig. 4, Additional files 8 and 17). In addition, 30 new QTLs were detected only by the conditional QTL analysis, suggesting that their effects on C18:3 might be masked by their effects on other traits (Additional files 4 and 16).

### Conditional unique QTLs for the five traits

Based on the conditional phenotypic values when C16:0, C18:0, C18:1, C18:2 and C18:3 were conditioned on each other, 49, 30, 48, 60 and 45 conditional consensus QTLs, respectively, were obtained in the six environments. These QTLs were integrated into 128 unique QTLs and distributed across all 19 chromosomes, except for A10 (Fig. 4, Additional files 8 and 18). Of these unique QTLs, 68 affected only one trait, while 60 had effects on two to five traits. Two conditional unique QTLs, *cuqA5–8* and *cuqA9–8*, affected the concentrations of all five FAs, and 11 QTLs influenced four different FA contents (Additional file 18). Moreover, 16 and 31 other QTLs were associated with three and two FA conditional phenotypic values, respectively.

### QTL comparison between unconditional and conditional mapping methodologies

In this study, QTLs detected by unconditional and conditional mapping analyses were compared. When QTLs identified by the two methods for the same trait had overlapping CIs, they were assumed to be identical. For C16:0, C18:0, C18:1, C18:2 and C18:3, 21, 21, 16, 21 and 22 consensus QTLs, respectively, were identified by the unconditional QTL mapping (Fig. 4 and Additional file 8). In contrast, many more QTLs were identified by the conditional QTL mapping analysis, including 30 for C18:0, 48 for C18:1, 60 for C18:2 and 45 for C18:3. Among them, 18, 16, 14, 19 and 15 QTLs were identified simultaneously by the two mapping analyses for C16:0, C18:0, C18:1, C18:2 and C18:3, respectively. In addition, the conditional QTL mapping analysis revealed 31 new QTLs for C16:0, 14 for C18:0, 34 for C18:1, 41 for C18:2 and 30 for C18:3 that could not be detected by the unconditional mapping.

In total, 71 unconditional unique QTLs and 128 conditional unique QTLs were obtained for the five FAs. A comparative analysis of the unique QTLs detected by the two methods revealed that 88.7% (63/71) of the unconditional unique QTLs were observed co-locating with conditional unique QTLs, and 65 additional unique QTLs were obtained when conditional QTL mapping was performed (Fig. 4, Additional files 8 and 19). The QTLs identified by multiple programs probably contained major genes associated with FA concentrations in *B. napus* seeds.

### Identification of potential candidate genes related to fatty acid synthesis

The 71 unconditional unique QTLs spanned a region of 220 cM, representing 10.85% of the total linkage map length. Further analyses showed that more than 5800 genes in *B. napus* were located in the QTLs’ CIs (data not shown). After a careful comparison with the FA synthesis genes in *Arabidopsis* [32], 150 of these genes were regarded as potential candidate genes affecting the five FA contents (Additional file 20). These candidate genes have roles in 22 different pathways, including plastidial FA synthesis, triacylglycerol (TAG) synthesis and lipid signaling. QTLs *uuqA5–6* and *uuqA5–7* were the most important, with large additive effects, that controlled the contents of the FAs, except C18:0, in most of the six environments. Two well-known FA synthesis genes, *BnaA05g26900D* (homologous gene of *FAD2*) in the TAG synthesis pathway and *BnaA05g27110D* (*GPAT5*) in the aliphatic suberin synthesis pathway, were found in the CI of *uuqA5–6*. One or more important genes affecting FA concentration may be in the CI of *uuqA5–7* based on the QTL mapping results. Four candidate genes, *BnaA05g28270D* (*CYTOCHROME P450*) in cutin synthesis, *BnaA05g28450D* (*SUGAR-DEPENDENT* 6) in mitochondrial phospholipid synthesis, *BnaA05g28620D* (*AT3G09920*) in lipid signaling and *BnaA05g28920D* (*PAH1*) in TAG synthesis and eukaryotic phospholipid synthesis (Additional file 20), were found in the CI of *uuqA5–7*. However, whether these genes or presently unidentified genes exerted great effects on FA concentrations, is still unclear.

In comparison, 65 new unique QTLs were detected by the conditional mapping analysis (Additional file 19). These QTLs covered 205.2 cM, with an equivalent physical region of 37.68 Mb. A total of 4633 *B. napus* genes were mapped on this region, and 164 of these genes were considered to be potential candidate genes (Additional file 21). A number of genes that had been confirmed to control FA synthesis were also assigned to conditional unique QTLs, such as *LPAAT4* (*BnaA07g21920D*) and *KASII* (*BnaA07g21940D*), which were assigned to the QTL *cuqA7–3*, *BC* (*BnaA09g48250D*) to the QTL *cuqA9–12*, *BCCP1* to the QTL *cuqC3–1* and *GPAT2* (*BnaC05g01190D*) to *cuqC5–1* (Additional file 21). Intriguingly, three regulatory factors underlying the QTLs’ CIs were also found: *FUS3* (*BnaA02g28280D*) was located in the CI of the QTL *cuqA2–4*, *LEC1* (*BnaC08g20060D*) was associated with *cuqC8–2* and *ASIL1* was in the CI of *cuqC6–2*. Thus, the combination of the two analytical methods identified promising functional genes that regulate FA biosynthesis.

## Discussion

The FA composition of *B. napus*’ seeds determines the oil’s nutritional and industrial values, and affects seed germination. Understanding the genetic control is a vital step in improving the oil. The FA levels of seeds are quantitative traits, and a large number of QTLs affecting FAs have been identified in *B. napus* [3–10, 33]. In this study, a high-density SNP map was used to map QTLs associated with five FAs on six environments, which allowed us to identify more reliable QTLs and candidate genes involved in regulating the composition of FAs in *B. napus*.

### A novel and major QTL located on A5 for FA composition

The synthesis pathways for the different FAs share the same basic substrates [22], resulting in close relationships. The QTL-level genetic analysis of the five FAs was performed based on phenotypic data from six different environments. There were 21, 21, 16, 21 and 22 consensus QTLs associated with C16:0, C18:0, C18:1, C18:2 and C18:3, respectively. Notably, two major consensus QTLs for C16:0 were detected on A5 and were located at the same positions as the major QTLs for C18:1, C18:2 and C18:3. These were integrated into two unique QTLs, *uuqA5–6* and *uuqA5–7* (Fig. 2). In previous studies, the major QTLs for C16:0 have been mapped on A8 and C3 [3, 5], and C2 and C8 [4]. For C18:0, the major QTLs were scattered throughout A8 and C3 [3, 5]. The major QTLs for C18:1 have been located on A3 [4, 7], A5 [8, 10], A8 [3, 5, 7], C3 [3, 4] and C8 [7], and the major QTLs for C18:2 have been mainly detected on A5 [8], A8 [3, 5, 7] and C3 [3–5, 7]. Additionally, the major QTLs for C18:3 have mainly been found on A3 [4], A4 [8, 10, 34], A7 [7], A8 [3], C3 [3, 4] and C4 [8, 10, 34]. Here, the two robust and steady major QTLs, *uuqA5–6* and *uuqA5–7*, provided powerful evidence that the A5 chromosome contains very important genes that influence the FA profile. Using GWAS, five consensus regions that mapped to the A2, A8, A9, C1 and C3 chromosomes were identified for seven fatty acids [20]. A cluster of loci on chromosome A5 (17.2–18.2 Mbp) was also identified for C18:1 and C18:2 using GWAS, with the strong candidate genes fatty acyl-ACP thioesterase B and *FAD5* [21]. This region was closed to the CI of *uuqA5–7*, but was not co-localized. Compared with QTLs detected in previous studies, *uuqA5–6* could be detected in different populations [8, 10], and *uuqA5–7* was potentially a new major QTL for FA. Liu et al. [35] identified a novel locus with the favorable allele BnA05-p22266340 on the A5 chromosome using the Brassica 60 K SNP array, which could increase the oil content of seeds by 1.5%–1.7%. However, they did not analyze which FAs were affected by the novel locus. Using the same Brassica 60 K SNP array, BnA05-p22266340 was mapped to 104.63 cM of the A5 in the AH map (Fig. 2) and was located in the CI of *uuqA5–7* (Additional file 5). Our study, together with previous observations, strongly suggested that one or more important genes underlying the CI of *uuqA5–7* could have important effects on C16:0, C18:1, 18:2 and C18:3, but no effect on C18:0.

### Three steady and valuable QTLs for marker-assisted selection (MAS)

Both *uuqA5–6* and *uuqA5–7* controlled C16:0, C18:1, C18:2 in all six environments and C18:3 in three and four environments, respectively, with mean additive effects of 0.14, − 3.09, 2.43 and 0.78, respectively, and 0.11, − 2.45, 1.95 and 0.56, respectively (Additional file 5). The unique QTL *uuqC4–9* was stably expressed in four or five environments with average additive effects of − 1.02, 0.83 and 0.66 for C18:1, C18:2 and C18:3, respectively (Additional file 5). Theoretically, when alleles came from the male parent ‘Holly’ in *uuqA5–6*, *uuqA5–7* and *uuqC4–9* synchronously, the C18:1 content increased ~ 13.12%, while the C16:0, C18:2 and C18:3 contents decreased by 0.5%, 5.21% and 2.1%, respectively. The three QTLs were much more important for quality breeding when performing MAS. These findings also suggested that it is rather hard to dramatically increase the C18:1 content without reducing the C18:2 and C18:3 contents in practical breeding programs. Additionally, 68 other unique QTLs were obtained in the present study, including 3 QTLs that controlled three traits and 16 QTLs that affected two traits, simultaneously. These QTL clusters provided promising genomic regions for MAS.

### Conditional QTLs were divided into four types

The unconditional QTL analysis showed that a number of QTLs affecting multiple traits, which was in accordance with the significant correlations based on phenotypic data. To evaluate possible genetic relationships among the five FAs at the individual QTL level, conditional mapping was performed using data of C16:0, C18:0, C18:1, C18:2 and C18:3 conditioned on each other, and 232 conditional consensus QTLs were obtained for the five FAs. Compared with the results of the unconditional mapping analysis, these conditional QTLs could be divided into four types: (1) QTLs that were detected only in the unconditional QTL analysis. Taking *ucqPA.A5–3* as an example, this QTL was repeatedly detected in all six experiments with a large additive effect for C16:0 (Additional file 1); however, when C16:0 was conditioned on C18:1, *ccqPA.A5–6* (co-localized with *ucqPA.A5–3*) failed to show a significant effect in any of the experiments (Additional file 6). This indicated that *ucqPA.A5–3*’s effect on C16:0 was entirely contributed by C18:1, and the genes underlying this locus simultaneously influenced the C16:0 and C18:1 contents; (2) QTLs that were detected in unconditional and conditional QTL analyses had similar additive effects. This phenomenon can be illustrated using the example of *ucqPA.A5–2*, which was the major QTL for C16:0 and was expressed in all six experiments (Additional file 1). The conditional QTL *ccqPA.A5–5*, which co-localized with *ucqPA.A5–2*, was still repeatedly detected for PA/ST in the six experiments with very similar genetic effect values (Additional file 6). These represent genes in the CI of *ucqPA.A5–2* that control the C16:0 content independently from the C18:0 content; (3) Although QTLs could be identified by both unconditional and conditional mapping, the assessment of the additive effects was dramatically changed by the different mapping methodologies. For instance, *ucqOL.A5–1* contributed 25.52% to the C18:1 content in the 13NJ environment with an additive effect of − 3.13 (Table 2), while *ccqOL.A5–2* (corresponding to *ucqOL.A5–1*) was still significant when the influence of C16:0 on C18:1 was excluded, and it explained 19.43% of PV with a reduced additive effect of − 2.55 (Additional file 12). This suggested that the effect of *ucqOL.A5–1* on C18:1 was partially due to the genetic effect on the C16:0 content; and (4) Additional QTLs were only detected by the conditional mapping method. These QTLs abounded in the present study, including 31, 14, 34, 41 and 30 for C16:0, C18:0, C18:1, C18:2 and C18:3 (Additional files 9, 11, 13, 15 and 17), respectively. The expression of these QTLs may have been completely suppressed by conditional traits; thus, their effects could only be detected when the influence of the conditional traits was eliminated. Together, these may better explain the genetic relationships among the five FAs at the individual QTL level compared with the correlations from the phenotypic data. Similar phenomena were also discovered in previous studies relating to oil and protein contents in *B. napus* [31], plant height, spike and internode lengths in wheat [29], and spike number, kernel number and thousand-kernel weight in wheat [36].

### The combination of unconditional and conditional QTL mapping is a powerful tool for dissecting the genetic basis of FA composition

The basic pathway of acyl-lipid metabolism is well characterized in *Arabidopsis* [22]. However, FA biosynthesis, modification and assembly into triacylglycerides are less well understood in *B. napus* because it has a more complex genomic structure than *Arabidopsis*. Brassica species and *Arabidopsis* have high degrees of sequence similarities and chromosomal collinearities [23, 24], and the possibility that genes that carry out the core biological processes will be orthologs. In fact, several orthologs encoding major enzymes involved in FA metabolism were mapped in *B. napus*, such as *FAD2* [8, 10, 37, 38], *FAD3* [8–10] and *fatty acid elongase 1* [39, 40]. Using a comparative genome analysis, 150 orthologs were obtained underlying the 71 unconditional unique QTLs (Additional file 20). The most important unique QTL *uuqA5–6*, which simultaneously affected C16:0, C18:1, C18:2 and C18:3, involved two well-known candidate genes. A candidate for this QTL was *FAD2* that encodes the enzyme that catalyzes the desaturation of C18:1 to C18:2, which was in accordance with previous studies [8, 10, 37, 38]. Another candidate was *GPAT5*, which exhibits a strong preference for sn-2 acylation and produces sn-2 lysophosphatidic acids as the major products of TAG synthesis [41]. Wang et al. [3] reported that *GPAT5* was associated with QTLs on A3, C3 and C5 in *B. napus*. Additionally, *uuqA5–7*, another major QTL was also detected on A5, explaining 16.48%, 24.42%, 25.98% and 17.60% of the PVs for C16:0, C18:1, C18:2 and C18:3, respectively, in different experiments. Among the genes underlying the CI of *uuqA5–7*, *PAH1* (*At3g09560*) encodes a phosphatidate phosphohydrolase, which is a key enzyme in the regulation of lipid synthesis and catalyzes the dephosphorylation of PA, yielding DAG and Pi [42]. This gene was the most likely candidate gene for *uuqA5–7*, but evidence that *PAH1* plays an important role in FAs biosynthesis in *B. napus* is lacking. In addition, *FAD3* was associated with QTLs *uuqA4–4*, *uuqC3–6* and *uuqC4–9* detected by two, two and three traits, respectively. *BCCP2* and *beta-CT*, subunits of the acetyl-CoA carboxylase complex in plastids, were assigned to *uuqA3–2* and *uuqC2–1*, respectively. Compared with the unconditional QTL analysis, 65 additional unique QTLs were obtained by conditional QTL mapping (Additional file 19), and 164 orthologs were in the CI of the new QTLs, including 6 and 17 genes involved in plastidial FA and TAG synthesis, respectively (Additional file 21). Three critical transcriptional factors, including *LEAFY COTYLEDON1* (*LEC1*, *AT1G21970*) [43], *FUSCA3* (*FUS3*, *AT3G26790*) [44] and *Arabidopsis* 6b-interacting protein 1-like 1 (*ASIL1*, *AT1G54060*) [22], regulating the oleosin gene’s expression and lipid accumulation were located in the CI of conditional unique QTLs, which could not be found in unconditional QTLs. *LEC1* was associated with *cuqC8–2*, *FUS3* was associated with *cuqC9–4* and *ASIL1* was associated with *cuqC6–2*. In a previous study, *LEC1* was assigned to A3, A8, A9 and C9, while *FUS3* was assigned to C7 [3].

## Conclusions

In this study, unconditional and conditional QTL mapping analyses were performed to decipher the genetic control of FAs in *B. napus*. Three pleiotropically unique QTLs (*uuqA5–6*, *uuqA5–7* and *uuqC4–9*) with important value for MAS were obtained from the unconditional mapping analysis, and *uuqA5–7* was a new major QTL for C16:0, C18:1, C18:2 and C18:3. A total of 232 conditional consensus QTLs were detected for the five FAs, and these QTLs were divided into four different types. Compared with the results of the unconditional mapping analysis, 65 new unique QTLs were detected when conditional QTL mapping was performed. The combination of two mapping methodologies provided useful information for MAS and the improvement of the FA composition of *B. napus*’ seeds.

## Methods

### Plant materials

A RIL population containing 189 lines and named the AH population, was used for QTL analyses of seed FA composition in the present study [16]. The two parents (‘APL01’ and ‘Holly’) were double low rapeseeds, with traces of erucic acid in the oil, but both had high levels of C18:1 (Fig. 1). The AH population was previously used for developing a high-density SNP map and for detecting QTLs associated with apetalous characteristics [16]. The genetic linkage map covered all 19 *B. napus* chromosomes of 2027.53 cM, with an average spacing of 0.72 cM between SNP-bins.

### Field trials and data collection

The AH population, as well as the two parents, were evaluated in six environments. The materials were planted in Dali for 1 year, September 2014 to May 2015, (14DL) and Yangling for 1 year, September 2015 to May 2016, (15YL) in Shaanxi Province, China; and in Nanjing for four consecutive years, September to May of 2012–2016, (12NJ, 13NJ, 14NJ and 15NJ, respectively) in Jiangsu Province, China. The experiment locations of DL and YL were the experiment bases of Hybrid Rapeseed Research Center of Shaanxi Province, and NJ was the experiment base of Jiangsu Academy of Agricultural Sciences. No specific permissions were required for the field trials. The field experiments were conducted based on Wang et al. [16]. At maturity, five representational plants were bulk harvested, and the seeds were used for FA measurements. The FAs profiled included C16:0 (Abbreviated as PA), C18:0 (ST), C18:1 (OL), C18:2 (LI) and C18:3 (LN). Bulked seed samples were analyzed by gas liquid chromatography using an Agilent 7890 series gas chromatograph (USA) in 12NJ and 13NJ environments according to Rücker and Röbbelen [45] and were determined by near infrared reflectance spectroscopy in 14DL, 14NJ, 15YL and 15NJ environments using a Foss NIRSystems 5000 according to the WinISI III manual’s instructions.

### Data analyses

Correlation analyses were implemented using SPSS 18.0 software (SPSS Inc., Chicago, IL, USA). Phenotypic correlation coefficients among the five FA compositions were calculated based on the traits for the two provinces. Unconditional phenotypic values were the mean value of the two replicates for each environment. The conditional values are estimated for the no-variation situation in the secondary trait, a method very similar to the estimation of adjusted values in a covariance analysis. The mixed model method in software QGAStation1.0 (http://ibi.zju.edu.cn/software/qga/) of the conditional analysis for quantitative traits was used to predict the conditional phenotypic values y(T1|T2) [27], where T1|T2 indicates trait 1 conditioned on trait 2 [31]. The default parameters of the model were used in the present study. For example, y(OL|LI) is the conditional phenotypic value of OL without the influence of LI. In previous studies, C16:0 showed highly significant correlations with C18:0, C18:1, C18:2 and C18:3 [3, 4, 21]. To investigate the genetic relationships among C16:0 and other four fatty acids, conditional QTL mapping analysis for C16:0 was also performed, although it is the first fatty acid type comparing to the other four types.

### QTL detection and meta-analysis

All five FAs were conditionally analyzed with each other in the six environments. Then, unconditional and the conditional phenotypic values for each trait collected in each environment were employed for QTL mapping analyses and named as unconditional QTLs and conditional QTLs, respectively, by the Windows QTL Cartographer 2.5 using the composite interval mapping model [46]. A stringent LOD threshold (2.8–3.1) of putative QTLs were determined by selecting 1000-fold permutation tests (*α* = 0.05), and these QTLs were termed ‘identified QTLs’. The QTL intervals were established by 2-LOD as approximately 95% QTL confidence intervals (CIs). A ‘two-round’ strategy of QTL integration was implemented to meta-analyze QTLs with overlapping CIs by the BioMercator V4.2 program [47]. In the first round, identified QTLs consistently expressed in different environments and with overlapping CIs for each trait were integrated into consensus QTLs. If a QTL that explained more than 20% of the phenotypic variation (PV) in at least one environment or more than 10% of the PV in at least two environments, then the QTL was defined as a major QTL [25]. In the second round, overlapping consensus QTLs for the different traits were integrated into pleiotropic unique QTLs [48]. The QTL nomenclature followed the method of Wang et al. [49] with certain modifications. Identified unconditional QTLs, were designated at the beginning with a prefix “*uiq*” (unconditional identified QTL), follow by the trait abbreviation, experiment code (1 = 12NJ, 2 = 13NJ, 3 = 14NJ, 4 = 14DL, 5 = 15NJ and 6 = 15YL) and linkage group (A1–A10 and C1–C9). If two or more identified QTLs were identified in a linkage group, a serial number was suffixed (e.g., *uiqPA6.A1–1*). Consensus QTLs were named with the prefix “*ucq*” (unconditional consensus QTL), trait abbreviation and linkage group (e.g., *ucqPA.A5–2*). Unique QTLs were named with the designation “*uuq*” (unconditional unique QTL) followed with the linkage group and the serial number of the QTL (e.g., *uuqA5–6*). For conditional QTLs, the name of identified QTLs, consensus QTLs and unique QTLs referred to the name of the corresponding unconditional QTLs, a designation beginning with the abbreviation “*ciq*”, “*ccq*” and “*cuq*”, respectively.

### Fatty acid composition’s candidate gene analysis

The *B. napus*’ reference genome sequence was released in 2014 [50], and the AH high-density SNP map had high degrees of chromosomal collinearities with the *B. napus* genome [16], allowing the convenient prediction of candidate genes underlying the QTL CIs within the *B. napus* genome. The probe sequences of SNPs on the unique QTL CIs were aligned to the *B. napus* reference genome using the method described by Liu et al. [35], and the genes underlying the corresponding *B. napus* genome region were hypothesized to be potential candidate genes. These *B. napus* genes were then used to search homologous genes in *Arabidopsis.* The whole-transcriptome RNA-seq was used to determine the expression values of acyl lipid-related genes from developing seeds removed from siliques of the *fae1* mutant at 7–8, 9–10 and 11–12 d after flowering in *Arabidopsis*, resulting in 1317 genes that associated with FA synthesis [32]. If the genes underlying the CIs of unique QTLs were homologous to one of the 1317 *Arabidopsis* genes, then they were considered as candidate genes.

## Additional files

Additional file 1: Unconditional identified QTLs and consensus QTLs obtained for C16:0 in six environments. (XLS 31 kb)

Additional file 2: Unconditional identified QTLs and consensus QTLs obtained for C18:0 in six environments. (XLS 29 kb)

Additional file 3: Unconditional identified QTLs and consensus QTLs obtained for C18:2 in six environments. (XLS 29 kb)

Additional file 4: Unconditional identified QTLs and consensus QTLs obtained for C18:3 in six environments. (XLS 27 kb)

Additional file 5: Unconditional unique QTLs obtained for the five fatty acid content in six environments. (XLS 47 kb)

Additional file 6: Conditional identified QTLs and consensus QTLs obtained for C16:0 in six environments. (XLS 50 kb)

Additional file 7: The locations of conditional consensus QTLs associated with fatty acids in the AH map. Conditional consensus QTLs distributed across the C subgenome are shown in this figure, and QTLs on the A subgenome are supplied in Fig. 3. The linkage groups are represented by vertical bars. The locus name and genetic distance are listed on the right and left of the corresponding chromosomes, respectively. The red regions on the linkage groups indicate that these regions harbor QTLs identified by the conditional QTL mapping analysis. Different colors denote different traits as indicated in the bar shown at the lower right corner of the picture. (TIFF 6380 kb)

Additional file 8: QTL comparison of the five fatty acid concentrations between unconditional and conditional mapping methodologies. QTLs located on the C subgenome are shown in this figure, and QTLs mapped to the A subgenome are provided in Fig. 4. Whole linkage groups are shown with black lines on the bottom, and molecular markers are labeled with short vertical bars. Consensus QTLs and unique QTLs obtained by the two methods are compared, and the QTL nomenclature is based on the descriptions in the Materials and methods (for example, *ucqPA* means unconditional consensus QTLs for C16:0). The black lines above the linkage groups show the QTL’ CIs, and the circles indicate the peak positions. (TIFF 2025 kb)

Additional file 9: QTL comparison between unconditional and conditional consensus QTLs for C16:0. (XLS 33 kb)

Additional file 10: Conditional identified QTLs and consensus QTLs obtained for C18:0 in six environments. (XLS 52 kb)

Additional file 11: QTL comparison between unconditional and conditional consensus QTLs for C18:0. (XLS 31 kb)

Additional file 12: Conditional identified QTLs and consensus QTLs obtained for C18:1 in six environments. (XLS 47 kb)

Additional file 13: QTL comparison between unconditional and conditional consensus QTLs for C18:1. (XLS 32 kb)

Additional file 14: Conditional identified QTLs and consensus QTLs obtained for C18:2 in six environments. (XLS 57 kb)

Additional file 15: QTL comparison between unconditional and conditional consensus QTLs for C18:2. (XLS 36 kb)

Additional file 16: Conditional identified QTLs and consensus QTLs obtained for C18:3 in six environments. (XLS 55 kb)

Additional file 17: QTL comparison between unconditional and conditional consensus QTLs for C18:3. (XLS 34 kb)

Additional file 18: Conditional unique QTLs obtained for the five fatty acid content in six environments. (XLS 81 kb)

Additional file 19: QTL comparison between conditional unique QTLs and unconditional unique QTLs for the five fatty acids. (XLS 48 kb)

Additional file 20: Potential candidate genes related to fatty acid synthesis that underlying the confidence interval of unconditional unique QTLs. (XLS 158 kb)

Additional file 21: Potential candidate genes related to fatty acid synthesis that underlying the confidence interval of additional conditional unique QTLs. (XLS 215 kb)

## Abbreviations

ASIL1: Arabidopsis 6b-interacting protein 1-like 1
DL: Dali in Shannxi Province, China
FA: Fatty acid
FAD2: Fatty acid dehydrogenase 2
FUS3: FUSCA3
GPAT5: sn-glycerol-3-phosphate acyltransferase 5
GWAS: Genome-wide association studies
LEC1: LEAFY COTYLEDON1
LI: C18:2
LN: C18:3
MAS: Marker-assisted selection
NJ: Nanjing in Jiangsu Province, China
OL: C18:1
PA: C16:0
PAH1: Phosphatidic acid phosphohydrolase 1
PV: Phenotypic variation
QTL: Quantitative trait locus
RIL: Recombinant inbred line
SNP: Single nucleotide polymorphism
ST: C18:0
YL: Yangling in Shannxi Province, China
