# Supplementary figures and images for: Unconditional and conditional QTL analyses of seed fatty acid composition in Brassica napus L
Source: BMC Plant Biol. 2018 Mar 23;18:49. doi: 10.1186/s12870-018-1268-7 (PMC5865336; doi:10.1186/s12870-018-1268-7)

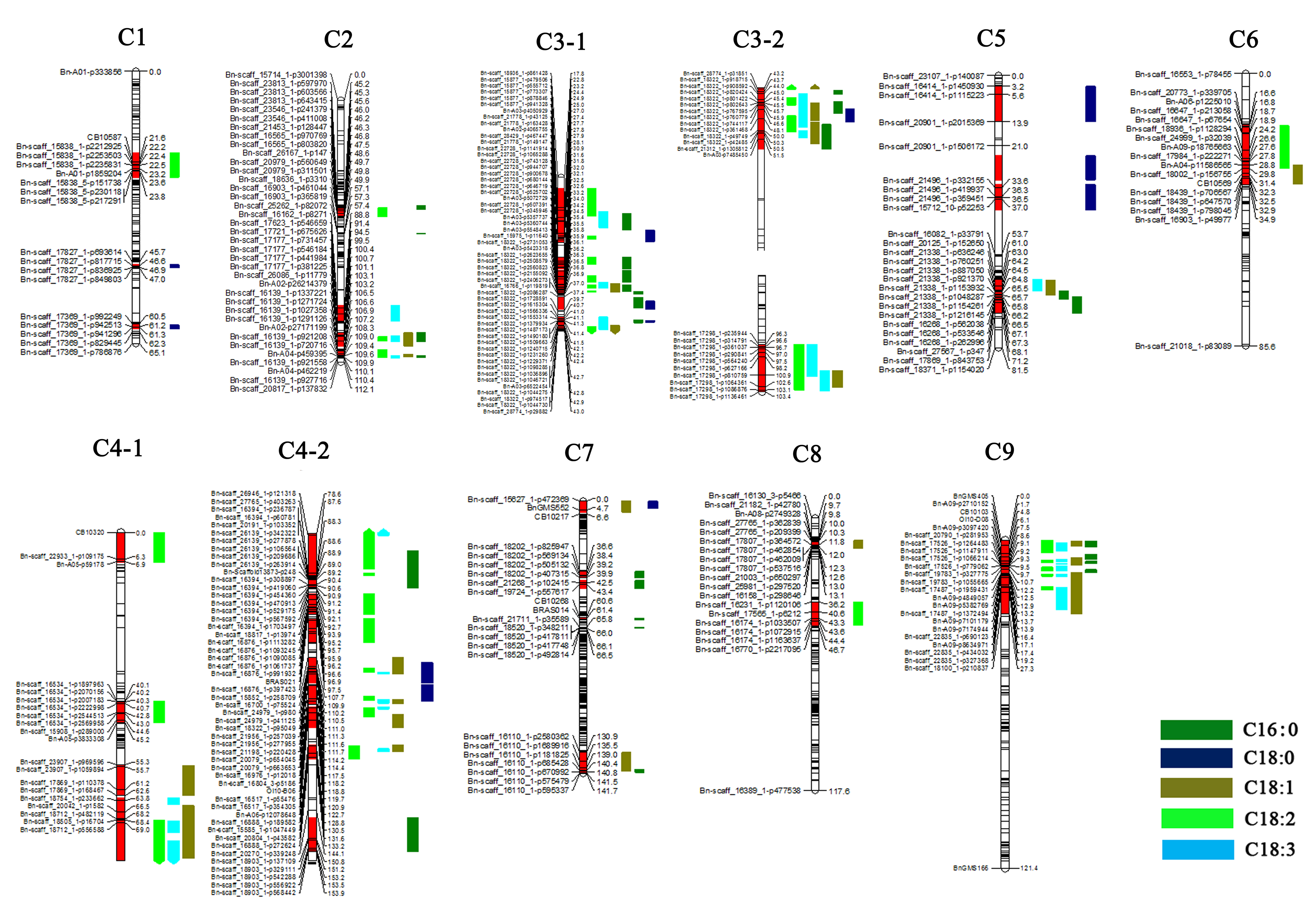

Supplement: Supplementary file 7 — The locations of conditional consensus QTLs associated with fatty acids in the AH map. Conditional consensus QTLs distributed across the C subgenome are shown in this figure, and QTLs on the A subgenome are supplied in Fig. 3. The linkage groups are represented by vertical bars. The locus name and genetic distance are listed on the right and left of the corresponding chromosomes, respectively. The red regions on the linkage groups indicate that these regions harbor QTLs identified by the conditional QTL mapping analysis. Different colors denote different traits as indicated in the bar shown at the lower right corner of the picture. (TIFF 6380 kb) [file 12870_2018_1268_MOESM7_ESM.tif]

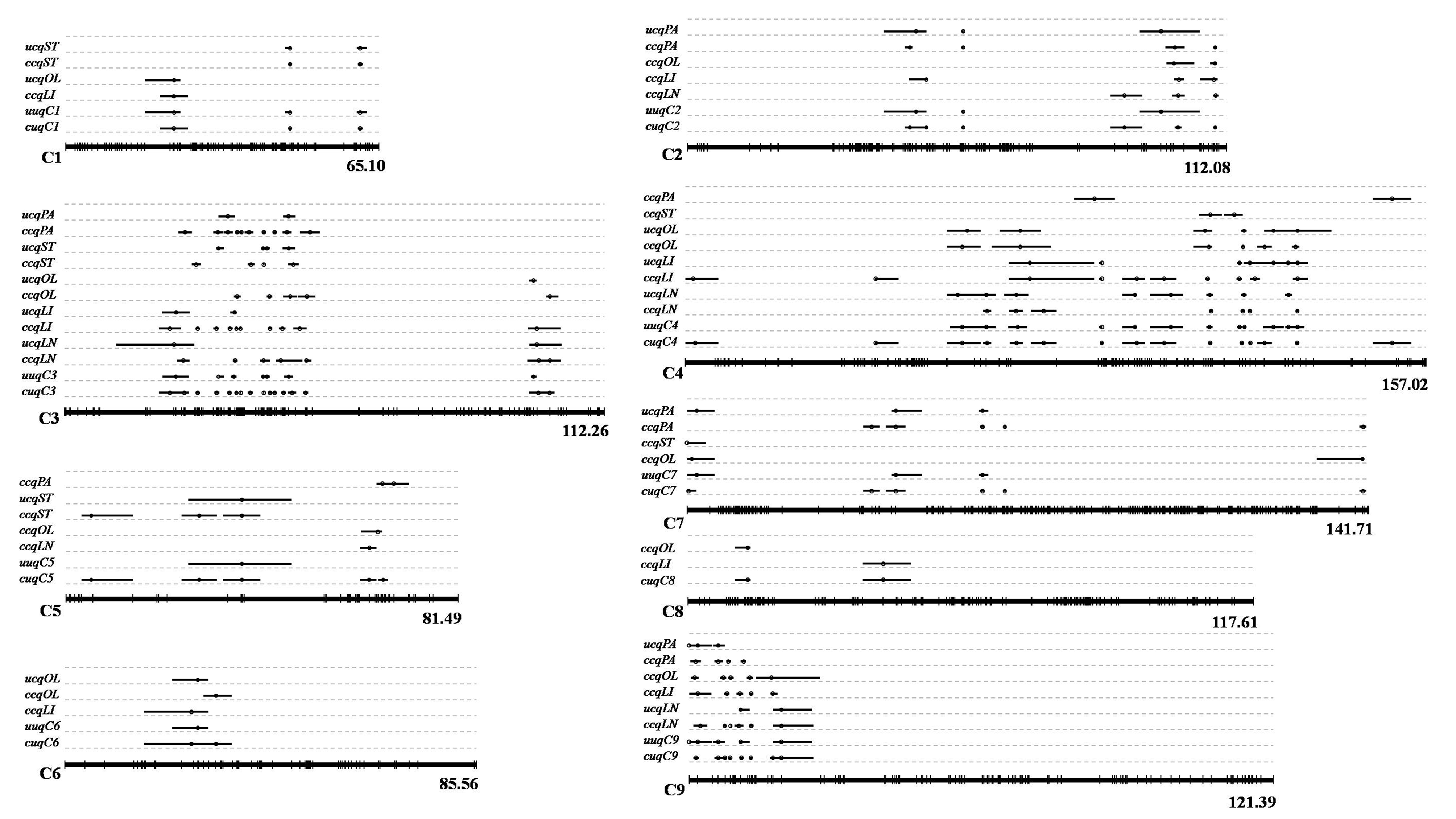

Supplement: Supplementary file 8 — QTL comparison of the five fatty acid concentrations between unconditional and conditional mapping methodologies. QTLs located on the C subgenome are shown in this figure, and QTLs mapped to the A subgenome are provided in Fig. 4. Whole linkage groups are shown with black lines on the bottom, and molecular markers are labeled with short vertical bars. Consensus QTLs and unique QTLs obtained by the two methods are compared, and the QTL nomenclature is based on the descriptions in the Materials and methods (for example, ucqPA means unconditional consensus QTLs for C16:0). The black lines above the linkage groups show the QTL’ CIs, and the circles indicate the peak positions. (TIFF 2025 kb) [file 12870_2018_1268_MOESM8_ESM.tif]
